# Network meta-analysis correlates with analysis of merged independent transcriptome expression data

**DOI:** 10.1186/s12859-019-2705-9

**Published:** 2019-03-15

**Authors:** Christine Winter, Robin Kosch, Martin Ludlow, Albert D. M. E. Osterhaus, Klaus Jung

**Affiliations:** 1 Institute for Animal Breeding and Genetics, University of Veterinary Medicine Hannover, Bünteweg 17p, Hannover, 30559 Germany; 2 Research Center for Emerging Infections and Zoonoses, University of Veterinary Medicine Hannover, Bünteweg 17p, Hannover, 30559 Germany

**Keywords:** Fold change, Gene expression, Meta-analysis, Network meta-analysis, Research synthesis

## Abstract

**Background:**

Using meta-analysis, high-dimensional transcriptome expression data from public repositories can be merged to make group comparisons that have not been considered in the original studies. Merging of high-dimensional expression data can, however, implicate batch effects that are sometimes difficult to be removed. Removing batch effects becomes even more difficult when expression data was taken using different technologies in the individual studies (e.g. merging of microarray and RNA-seq data). Network meta-analysis has so far not been considered to make indirect comparisons in transcriptome expression data, when data merging appears to yield biased results.

**Results:**

We demonstrate in a simulation study that the results from analyzing merged data sets and the results from network meta-analysis are highly correlated in simple study networks. In the case that an edge in the network is supported by multiple independent studies, network meta-analysis produces fold changes that are closer to the simulated ones than those obtained from analyzing merged data sets. Finally, we also demonstrate the practicability of network meta-analysis on a real-world data example from neuroinfection research.

**Conclusions:**

Network meta-analysis is a useful means to make new inferences when combining multiple independent studies of molecular, high-throughput expression data. This method is especially advantageous when batch effects between studies are hard to get removed.

**Electronic supplementary material:**

The online version of this article (10.1186/s12859-019-2705-9) contains supplementary material, which is available to authorized users.

## Introduction

Network meta-analysis has been widely used for aggregating results of clinical trials to make direct and indirect inferences about treatment effects, and several methodical concepts for network meta-analysis have been proposed [1–3]. Published examples of network meta-analysis are for example the comparison of the efficacy of different treatments against each other [4], the comparison of different therapies [5], or the study of safety of different drugs [6]. In contrast to ‘traditional’ meta-analysis which aggregates studies on the same study question, network meta-analysis also involves studies on different study questions which are linked by pairwise same treatment groups. Treatment comparisons that have not been studied in the original studies can indirectly be made within the network meta-analysis. Thus, inferences about group comparisons which are not linked within the network of study groups from the original studies are possible. While ‘traditional’ meta-analysis has already been used to merge the results of high-dimensional gene expression studies from microarray or RNA-seq experiments, and this topic has also been elaborated methodically [7–9], the relatively new methodology of network meta-analysis has not been considered for such data so far. Examples of ‘traditional’ meta-analysis of high-dimensional expression data are for example the identification of genes differentially expressed in cancer [10] or in neurological tissues [11, 12].

The aim of this work is to compare network meta-analysis as a tool for indirect inferences with the analysis of merged gene expression data. Since most journals in the area of high-dimensional expression data demand submitting authors to deposit their original data in public repositories such as Gene Expression Omnibus (GEO) [13] or ArrayExpress (AE) [14] alternatives to meta-analysis and network meta-analysis have opened up: the direct merging and subsequent joint analysis of the original data. Merging of original data can also be an approach of making indirect comparisons. However, merging becomes difficult if the data was taken using devices from different manufacturer or even different technologies. Problems in data merging may for example arise when expression data in some studies were taken by means of DNA microarrays as continuous fluorescence values [15] and by means of RNA-seq as read counts [16] in other studies. In some cases, batch effects between different types of expression data can be removed [17, 18]. However, even after applying a batch effect removing step onto the merged data false discoveries may occur as was shown by [19]. Therefore, merging of results in form of meta-analysis appears to be advantages in such cases, meaning that meta-analyses should be preferred over data merging strategies. Hereupon, the question arises how comparable indirect inferences from network meta-analysis and from the analysis of merged data are.

In this article, we evaluate the possibility of indirect group comparisons using either the strategy of data merging or of network meta-analysis. Specifically, we study how strong the lists of differentially expressed genes detected in indirect group comparisons by either type of analysis differ. Furthermore, we study how strong the indirect fold changes of genes determined by the two ways of analysis are correlated, and how strong they are correlated to the true fold changes. After briefly describing the approaches of network meta-analysis and the alternative analysis variant based on merged data sets, we demonstrate the benefits and limitations of either approach in a simulation study and on a data example of high-dimensional gene expression data from infection research.

## Methods

**Fig. 1:**
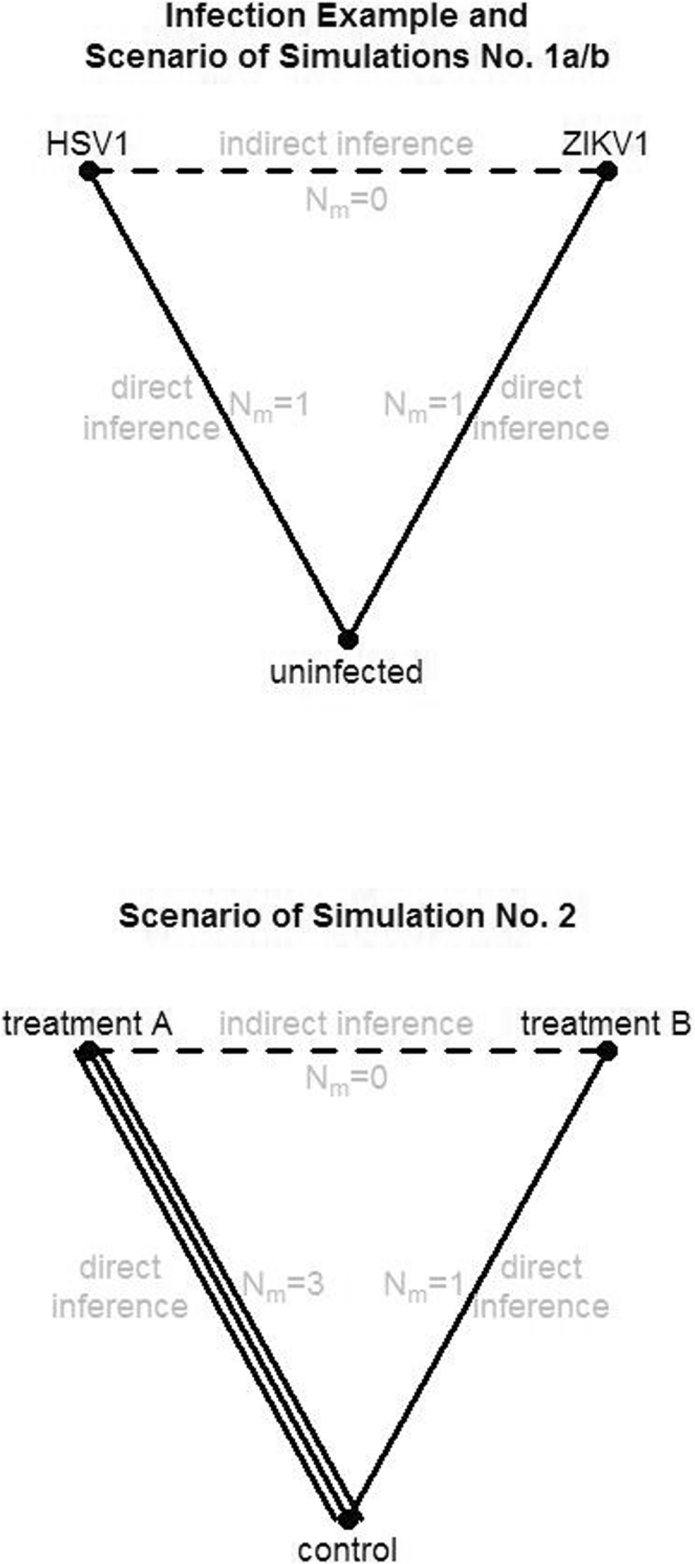
Schemes of study networks. Networks were either simulated or represent the infection example. Top: two studies are connected by a similar control group. (This scenario is evaluated in simulations no. 1a and no. 1b and by the infection example.). Bottom: the edge representing the comparison between treatment A and control is supported by three independent studies (This scenario is evaluated in simulation no. 2.)

Consider a study network with *n* different experimental groups. Let further *m* denote the number of possible pairwise group comparisons in this network. Thus, a graph is formed with *n* nodes and *m* edges. In practice, not all *m* edges will be covered by direct study internal comparisons. In this case, *m*_*direct*_≤*m* denotes the number of existing comparisons for which effect estimates are available directly from at least one study. One goal of the network meta-analysis is to obtain estimates for the non-existing *m*_*indirect*_=*m*−*m*_*direct*_ comparisons. The study networks depicted in Fig. 1 consist of *n*=3 nodes and *m*_*direct*_=2 directly available comparisons, while for *m*_*indirect*_=1 pair of study groups no direct comparisons exist from the original studies. Thus, the whole study network consists of *m*=3 edges. In the study network at the bottom of Fig. 1, the comparison of treatment A versus control is supported by three independent studies. Thus, the number of available independent comparisons can be even larger than *m*_*direct*_. We therefore introduce $m^{\prime }_{direct}\geq m_{direct}$ as the number of available independent comparisons in the network.

Differential expression analysis can either be performed on the $m^{\prime }_{direct}$ available individual studies so that results can be merged in a network meta-analysis. Alternatively, differential expression analysis can be performed on the merged data. Both variants allow for direct and indirect inferences.

### Differential testing

As method for differential testing between each pair {*k,k*^′^} of experimental groups (*k*≠*k*^′^;*k,k*^′^=1,...,*n*) we use the linear models implemented in the R-package ‘limma’ [20]. After fitting this model to the data, we obtain for each gene *g* (*g*=1,...,*G*) the estimated regression coefficient and its related standard error from the result object from the ‘eBayes’ function of the ‘limma’-package:

1 $$ \hat\beta_{g}\hspace{.5cm}\text{and}\hspace{.5cm}SE\left(\hat\beta_{g}\right).  $$

In these linear models, the regression coefficients can be interpreted in the sense of the log fold change of a gene between two experimental groups. Besides, test results in form of a *p*-value per gene are procuced, of course. Fold changes, standard errors and *p*-values can then be used in the network meta-analysis to bring together the results of the individual studies and also to make indirect comparisons.

### Network meta-analysis

To estimate regression coefficients and their standard errors within the network of comparisons (direct as well as indirect comparisons) we employ the method proposed by [2] which we briefly sketch in the following and refer the reader to this publication for further details. The calculations of the network meta-analysis are done separately for each gene *g* (*g*=1,...,*G*). Here, *G* is the number of genes jointly studied in all independent studies. Genes for which the expression measurements are not available in all studies are excluded from the analysis. To determine in this network the log fold change of gene *g* and its standard error related to the comparisons of all *m* pairs of groups {*k,k*^′^}(*k*≠*k*^′^,and*k,k*^′^=1,...,*n*), a (*mdirect*′×*mdirect*′) weight matrix **W** is constructed first, with diagonal elements $1/SE(\hat \beta)^{2}$ and with all other entries being equal zero. With this weight matrix, comparisons with a high standard error get less weight in the network. Furthermore, the regression coefficients $\hat \beta _{g}$ from the individual comparisons are stored in the vector **x**. Next, an (*mdirect*′×*n*) matrix **B** is constructed where each row represents one of the $m^{\prime }_{direct}$ available comparisons, and where the connections of the nodes to each other are represented. Therefore, in each row of **B**, a 1 is put in the column related to the node of experimental group *k* and a -1 one is put in the column related to the other group *k*^′^ of the available comparison represented by this row. All other elements are zero. Thus, matrix **B** shows for which pairs of experimental groups, results of differential expression analysis are available from the original studies. Using the matrices **W** and **B**, a Laplacian matrix as used in graph theory and its Moore-Penrose inverse are calculated as follows:

2 $$ \mathbf{L}=\mathbf{B}^{T}\mathbf{WB}, \text{ and } \mathbf{L}^{+}=(\mathbf{L}-\mathbf{J}/n)^{-1}+\mathbf{J}/n,  $$

where **J** is an (*n*×*n*) matrix of ones. The variances of the log fold changes in the network meta-analysis can then be determined by the (*n*×*n*) matrix **R** with entries

3 $$ \mathbf{R}_{k,k'}=\mathbf{L}^{+}_{k,k}+\mathbf{L}^{+}_{k',k'}-2\mathbf{L}^{+}_{k,k'}.  $$

Note that *R* is symmetric, i.e. $\phantom {\dot {i}\!}R_{k,k'}=R_{k',k}$. The standard errors for each comparison in the network meta-analysis are then given by $\sqrt {\mathbf {R}}$.

In order to calculate estimates of the direct log fold changes in this network, stored in vector **v** of length $m^{\prime }_{direct}$, the following equation is used:

4 $$ \mathbf{v}=\mathbf{BL}^{+}\mathbf{B}^{T}\mathbf{Wx}.  $$

In the case that $m^{\prime }_{direct}=m_{direct}$, the elements of **v** are equal to the input fold changes stored in *x*. In cases where $m^{\prime }_{direct}>m_{direct}$, the elements of **v** for network edges which are supported by multiple studies are a summary of the fold changes from these studies. The fold changes for the indirect comparisons can be obtained by a subtraction procedure between the elements of **v**. This subtraction procedure is detailed by the example code provided within [2] (cf. three fold for-loop to construct the matrix ‘all’ in their example code). To perform these calculations in our simulations and in the analysis of the infection data, we employ the R-package ‘netmeta’ that provides the implementation of the methods by [2].

Example R-code that shows how to use the ‘limma’-results in the package ‘netmeta’ is provided as supplementary material (Additional file 1).

### Batch effect removal in merged data sets

A regular problem when merging data from different studies are batch effects. Therefore, we base our simulation study on a gene expression model that includes additive and multiplicative batch effects [17]. This model was recommended as a results of a systematic comparison by [18]. We refer to a further comparison of methods for batch effect removal in the discussion section of this work. In this model, the gene expression level of gene *g* in group *j* and study *i* is drawn by

5 $$ Y_{ijg}=\alpha_{g} + \beta_{gj} + \gamma_{ig} + \rho_{ig}\epsilon_{ijg},   $$

where *α*_*g*_ and *β*_*gj*_ are the overall and the group specific expression level, respectively. The components *γ*_*ig*_ and *ρ*_*ig*_ are an additive and multiplicative batch effet, respectively, and *ε*_*ijg*_ is the overall error. Estimation and removal of these types of batch effects are implemented in the ‘ComBat’ function of the R-package ‘sva’.

## Results

To evaluate network meta-analysis of transcriptome profiles and to compare the results with the analysis of merged data sets, we ran a simulation study and applied the methods to an example from neuroinfection research. All simulation scenarios were first performed using 500 runs and then repeated with 1000 runs which led to the same conclusions. Therefore, the authors considered 1000 runs a appropriate choice.

### Simulation study

Simulation no. 1a represents two studies on two different diseases (A and B), each study involving samples from diseased individuals and from healthy controls. In practice, a researcher would usually be interested in a comparison of two diseases from a similar area (e.g. different cancers or different infectious diseases). While the individual studies provide the direct comparison between samples from the disease group versus control samples, data merging or network meta-analysis can be used to make the indirect comparison of the samples from the two disease groups (Fig. 1). The comparison of the transcriptome expression data from the disease groups could provide insights about their differences, e.g. which genes are highly expressed under disease A but not under disease B. Assuming, alternatively, not a scenario with diseases but with different treatments (where the control group represents untreated samples) the indirect comparison of the different treatment samples could uncover which genes are influenced by treatment A but not by treatment B.

In the simulation, the parameters of the model specified by Eq. (5) were mainly drawn from the normal distribution except for the multiplicative batch effect which was drawn from the inverse Gamma distribution (Table 1). Using the inverse Gamma distribution was also proposed by [17] to obtain values distributed around 1. Hence, for most genes, the multiplicative effect is rather weak. For both studies, different values of the distribution parameters were chosen for the batch effects. Note also, that the term for the fold change, *β*_*gj*_, was set to zero for the control groups. In total, we simulate data for *G*=100 genes which is enough, here, to compare ranking lists from differential expression analysis. Sample sizes per group were chosen as *n*_1_=*n*_2_=10 in this simulation.

**Table 1:** Setting of simulation parameters

Group/group	*α*_*g*_	*β*_*gj*_	*γ*_*ig*_	*ρ*_*ig*_	*ε*_*ijg*_
Study 1: Control	$\mathcal {N}(0, 1)$	0	$\mathcal {N}(0, 1)$	*InvGamma*(1,1)	$\mathcal {N}(0, 1)$
Study 1: Disease A	$\mathcal {N}(0, 1)$	$\mathcal {N}(0, 1)$	$\mathcal {N}(0, 1)$	*InvGamma*(1,1)	$\mathcal {N}(0, 1)$
Study 2: Control	$\mathcal {N}(0, 1)$	0	$\mathcal {N}(2, 1)$	*InvGamma*(1,2)	$\mathcal {N}(0, 1)$
Study 2: Disease B	$\mathcal {N}(0, 1)$	$\mathcal {N}(0, 1)$	$\mathcal {N}(2, 1)$	*InvGamma*(1,2)	$\mathcal {N}(0, 1)$
Study 3: Control	$\mathcal {N}(0, 1)$	0	$\mathcal {N}(0, 1)$	*InvGamma*(1,1)	$\mathcal {N}(0, 1)$
Study 3: Disease A	$\mathcal {N}(0, 1)$	$\mathcal {N}(0, 1)$	$\mathcal {N}(0, 1)$	*InvGamma*(1,1)	$\mathcal {N}(0, 1)$
Study 4: Control	$\mathcal {N}(0, 1)$	0	$\mathcal {N}(0, 1)$	*InvGamma*(1,1)	$\mathcal {N}(0, 1)$
Study 4: Disease A	$\mathcal {N}(0, 1)$	$\mathcal {N}(0, 1)$	$\mathcal {N}(0, 1)$	*InvGamma*(1,1)	$\mathcal {N}(0, 1)$

Simulation nos. 1a and 1b involve studies 1 and 2, only, while simulation no. 2 involves all four studies

**Fig. 2:**
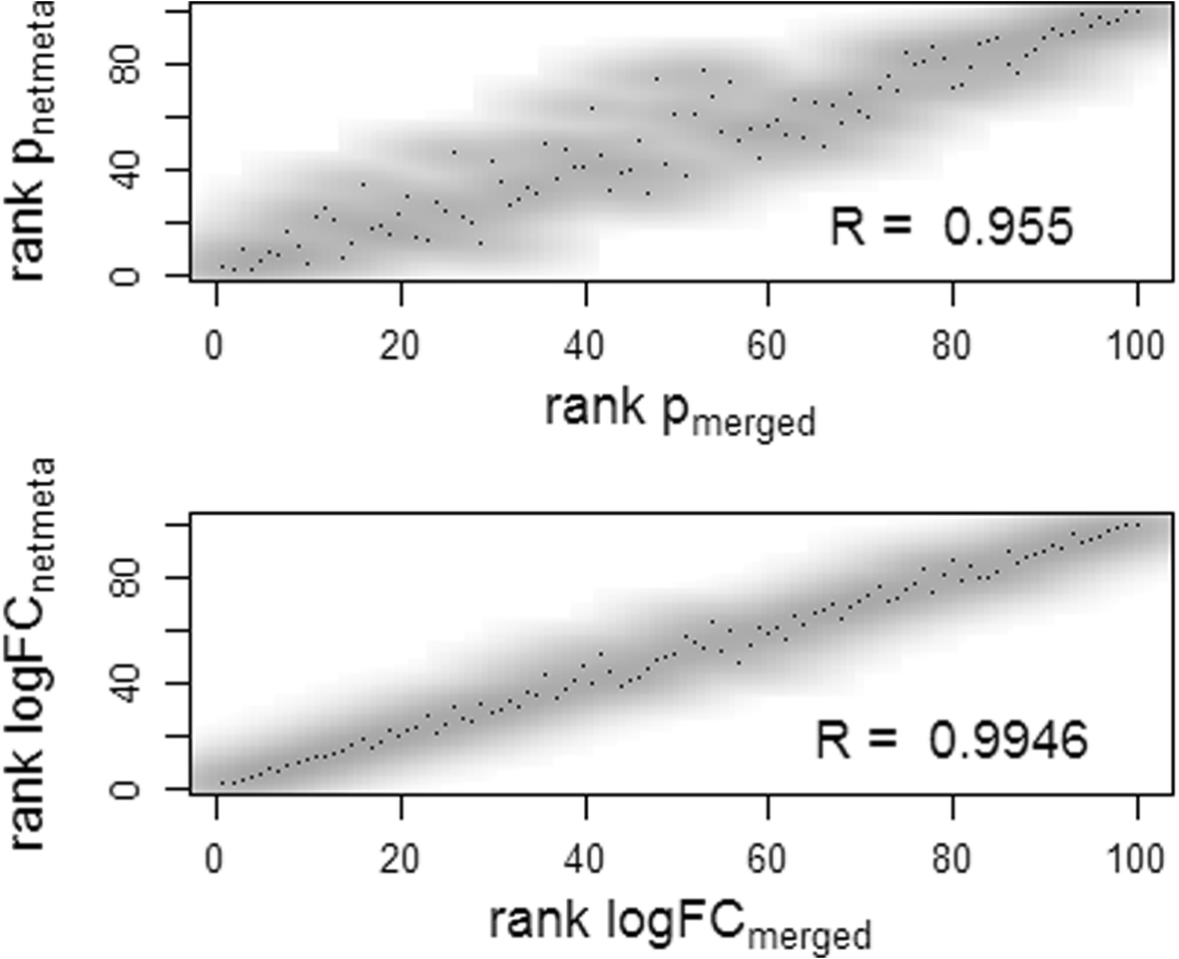
Correlation between results of data merging versus results of network meta-analysis. Smoothed scatterplots representing the ranks of *p*-values (top) and of log fold changes (bottom), respectively, resulting from network meta-analysis versus the results from the analysis of merged data in the simulation of two independent studies (Simulation no. 1a). The plots represent the results from 1 of 1000 simulation runs

Comparing in simulation no. 1a the ranks of *p*-values and ranks of log fold changes from the network meta-analysis versus those from the merged data analysis, high correlations can be observed (Fig. 2). The results of both analysis variants would therefore lead to similar biological conclusions. If we look at the true simulated fold changes, *β*_.1_−*β*_.2_, and correlate them with either the fold changes from the network meta-analysis or from the merged data analysis, again no large differences between the two analysis variants could be observed. Taken from 1000 simulation runs, the mean (+/- standard deviation) correlation between the true fold changes and those from the network meta-analysis or from the merged data was 0.74 +/- 0.18 each (Additional file 2).

**Fig. 3:**
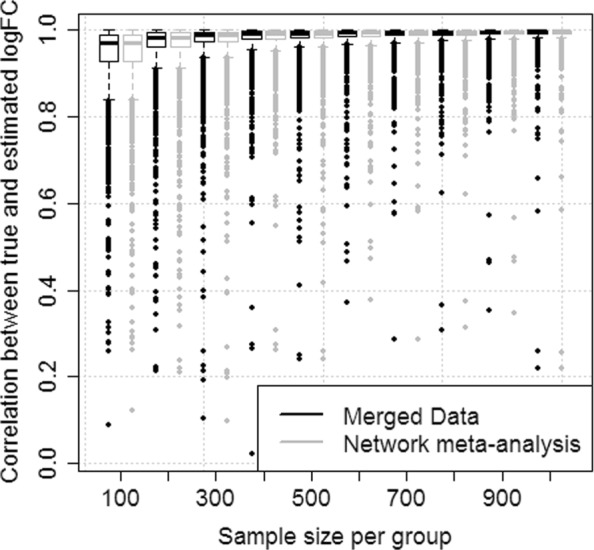
Precision of fold change estimation in a simple scenario. Boxplots representing the correlation between true and estimated logFC versus sample size per group observed in the analysis of merged data and in network meta-analysis. 1000 simulation runs of two independent studies were performed per sample size (Simulation no. 1b). Both analysis variants show nearly the same correlation which increases with increasing sample sizes

In order to study how the correlation between the true fold changes and those from either network meta-analysis or from the merged data analysis changes when sample sizes are increased, the simulation scenario was extended with sample sizes per group being increased from *n*_1_=*n*_2_=100 to *n*_1_=*n*_2_=1000 by steps of 100 (simulation no. 1b). Again, 1000 runs were performed for each value of the sample sizes. In this simulation, the correlation increases when the sample size per group was increased (Fig. 3). Still, no relevant differences in the correlation can be seen between the two analysis variants, i.e. for each sample size the corresponding two boxplots are nearly identical.

**Fig. 4:**
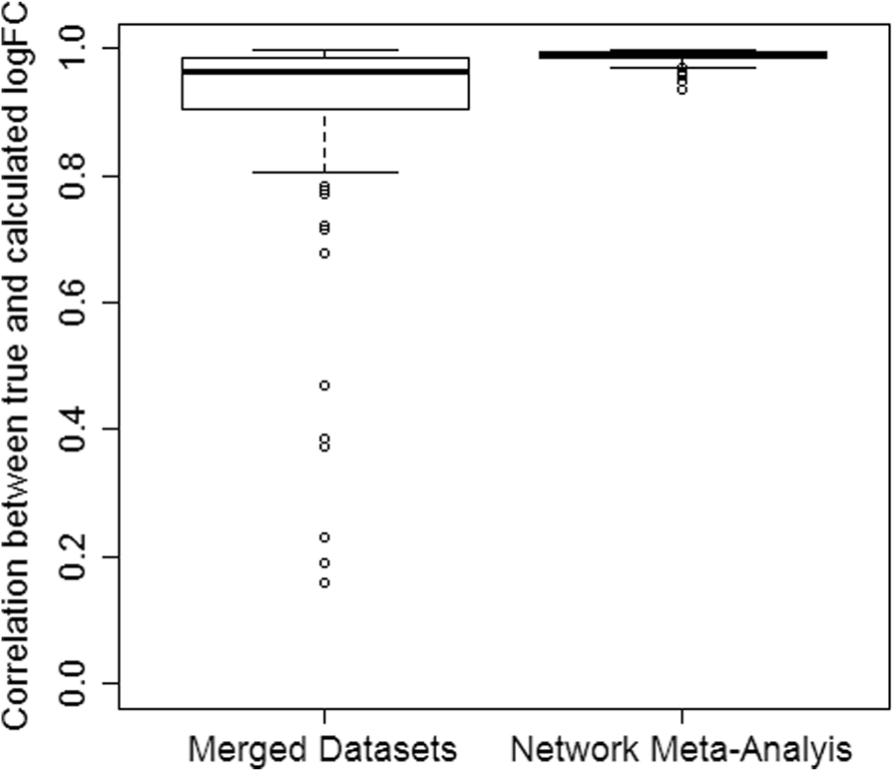
Precision of fold change estimation in more complex scenarios. Correlation between true and estimated logFC observed in the analysis of merged data and in network meta-analysis from 1000 runs of simulation scenario no. 2., where one edge of the network is represented by multiple independent studies

Network meta-analysis also allows that an edge of the network is supported by multiple studies (Fig. 1 bottom). In simulation no. 2, we generated data from three independent studies to support the comparison between treatment A and control, and data from one study to support the comparison between treatment B and control. In this scenario, the correlation between true and calculated logFC was overall higher when using network meta-analysis than in the analysis of the merged data (Fig. 4).

### Examples: transcriptome expression profiles in neuroinfectious diseases

Our real world example involved transcriptome expression profiles from ZIKA virus (ZIKV) infected neural progenitor cells [21] as well as expression profiles of differentiated NT-neurons infected with herpes simplex virus 1 (HSV1). No journal publication is available for the latter study. Both data sets were selected from GEO with accession numbers GSE80434 (African ZIKVM and mock infected samples only) and GSE24725, respectively. Furthermore, both studies follow a two group design with the infected cells compared to control samples. ZIKV is a mosquito-borne Flavivirus, first discovered in 1947 in Uganda [22]. HSV1 belongs to the class of Herpesviridae and is transmitted by direct contact. The capacity of both viruses to infect neural tissues following initial systemic virus spread means that a network meta-analysis can be helpful to identify genes that show a different expression in hosts infected by either virus [23]. The intersection of both studies was *G*=7912 genes that were subjected to the joint analysis. In order to compare the expression profiles of ZIKV and HSV1 infected neural cells we first merged both data sets, performed the batch effect removal and finally differential expression analysis. We denote the resulting *p*-values and log fold changes by *p*_*merged*_ and *logFC*_*merged*_, respectively. As second analysis variant, we performed network meta-analysis obtaining *p*_*net*_ and *logFC*_*net*_, respectively.

**Fig. 5:**
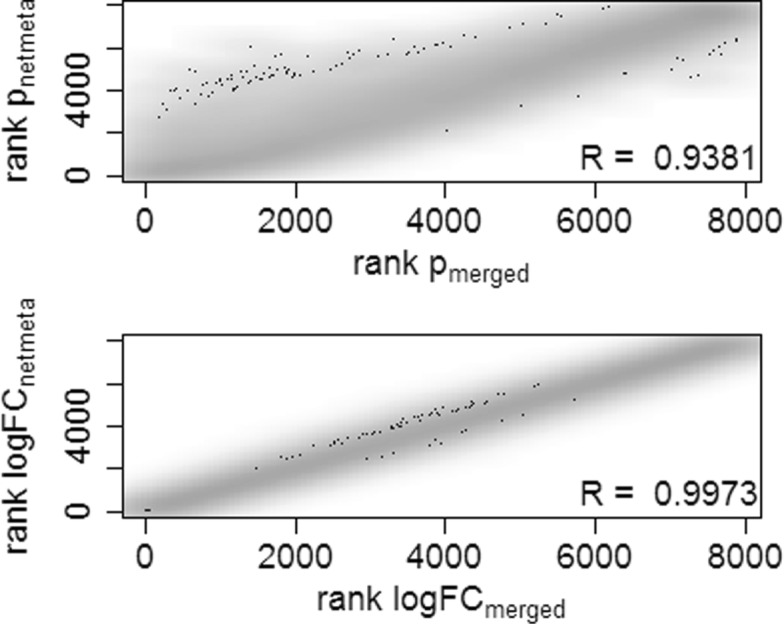
Correlation of results in infection data set. Smoothed scatterplots representing the ranks of *p*-values (top) and log fold changes (bottom), respectively, resulting from network meta-analysis versus the results from the analysis of merged ZIKV and HSW1 data sets

In general, the order of the *p*-values and log fold changes in both analysis variants were highly but not perfectly correlated (Fig. 5). Thus, the top selected genes can differ between the two strategies, and biological conclusions can vary. Variations in the biological interpretation from both analysis strategies will be discussed in the last chapter.

In addition to the differential expression analysis, we studied how gene set enrichment analysis changes when using either merged data analysis or network meta-analysis. Therefore, ranked gene lists resulting from differential expression analysis were subjected to GO term enrichment analyses. In total 4860 GO terms were analysed. Based on the merged data analysis, 43 GO terms were significantly enriched among the differentially expressed genes between ZIKV and HSV1, while 67 GO terms were selected when using network meta-analysis. The overlap of these two sets included 13 GO terms that would contribute to the biological interpretation regardless of the type of analysis. Again, the commonalities and differences in biological interpretation will be discussed in the last chapter.

## Discussion

### Differences in biological interpretation

The analyses of the infection data by data merging and network meta-analyis, respectively, have shown commonalities and differences in the results. This can have consequences on the biological interpretation as will be demonstrated in the following.

In general, among the top 10 genes selected with both analysis variants (Table 2) are genes with diverse functions in cell recruitment, apoptosis or neuronal development. Some of these genes were already described in connection with the development of other neuropathic diseases, in particular with Alzheimer’s, Parkinson’s and Huntington’s Disease.

**Table 2:** Top 10 differentially expressed genes (i.e., with the smallest *p*-values), selected from either merged data sets (left) and network meta-analysis (right), respectively

Rank	Merged analysis	Network meta-analysis
1	**COX7B**	**RHO**
2	**CXCR3**	**LTB**
3	**LTB**	**CXCR3**
4	**RHO**	**COX7B**
5	**TNFAIP2**	TPO
6	SF1	SLC39A2
7	ENO1	HAL
8	SLC4A1	**TNFAIP2**
9	**PNMA1**	**PNMA1**
10	H1FX	MFN2

Bold names indicate 6 genes that appear in both top 10 lists

Looking at the 6 genes that occur in both top 10 lists, CXCR3 is expressed on activated T-lymphocytes, natural killer cells and on B-lymphocyte subsets and mediates T-cell migration into inflammatory areas of the nervous system during viral infection [24, 25]. Furthermore, CXCR3-deficient mice showed an increased mortality rate (associated with higher viral load) after West Nile Virus (WNV) or dengue virus infection [26, 27] that can also lead to neuropathic diseases. In contrast, an elevated level of viral clearance was observed during HSV-1 encephalitis in CXCR3-deficient mice resulting in reduced clinical signs and decreased mortality [28, 29]. CXCR3 activation lead to transactivation of pro-inflammatory genes, and initiation of apoptosis in neurons. To prevent neuronal cell death during WNV Encephalitis, WNV-infected cells induce TNF *α*-regulated signaling pathways which result in down regulation of CXCR3 [30]. COX7B is one of the small, nucleus-encoded subunits of cytochrome c-oxidase, the terminal complex in the mitochondrial respiratory chain. The small subunits have regulatory functions and play an essential role in complex assembly [31]. Furthermore, mutations lead to microcephaly, indicating a role for COX7B in brain and eye development [32]. Expression changes of COX7B have also been described during the development of neurodegenerative diseases [23, 33, 34]. Anti-PNMa1 autoantibodies can be found in patients with paraneoplastic neurological disorders [35] in connection with brainstem or limbic encephalitis, hypothalamic disorder and dementia. Furthermore, PNMA1 expression is also increased in apoptotic neurons, although the underlying mechanism is poorly understood [36]. Lymphotoxin B (LTB) is a type II membrane protein encoded by the LTB gene and plays a key role during lymph node development, LTB gene deletion in mice leads to a lack of peripheral lymph nodes and Peyer’s patches [37]. LTB only binds its receptor LTBR, leading to NF *κ*B activation and cell death [38, 39]. With respect to ZIKV and HSV-1, these 6 genes could play a similar role.

Among the top 10 genes selected by the analysis of the merged data are ENO1, H1FX, SF1, SLC4A1, which only occur in the network meta-analysis from rank 469 and below, and would probably not be considered in a biological interpretation of the results. ENO1 catalyzes the penultimate step in glycolysis, but is also involved in regulation processes, such as inflammatory cell recruitment [40] and tumor suppression [41]. The protein interacts with ZIKV non-structural proteins and is able to influence cell proliferation and differentiation [42, 43]. H1FX belongs to the histone H1 family. H1 linker histones bind the nucleosomal core particle around the DNA entry and exit sites and stabilize the chromatin structure. In this way, H1 proteins are involved in transcriptional regulation, but also play a role in cell proliferation and differentiation. All H1 variants have the same general structure, but differ in their functions [44]. SLC4A1 is a chloride-bicarbonate exchanger expressed in erythrocytes and intercalated cells of renal collecting ducts. Mutations of SLC4A1 have been described associated with distal renal tubular necrosis and haemolytic anemia [45]. Little is known about SF1 in connection with neuroinfection.

In contrast, the top10 genes selected by network meta-analysis included HAL, MFN2, TPO, SLC39A2 which also occur among the top20 list obtained from the analysis of the merged data. Therefore, these 4 genes would eventually be regarded in the interpretation of both analysis results. Mitofusin 2 (MFN2) GTPase is a mitochondrial membrane protein that is also crucial in mitochondria metabolism [46]. Furthermore, MFN2 is involved in activation of the inflammasome in macrophages during virus infection [47]. The loss of MFN2 lead to an enhanced virus-induced synthesis of IFN *β* and decreased viral reproduction [48]. Thyroid Peroxidase (TPO) is expressed in the thyroid gland and is essential for thyroid hormonogenesis. Nevertheless, TPO promotor also contains a specific NF *κ*B binding site, leading to transactivation after (LPS) stimulation [49].

In the gene-set enrichment analysis 13 GO terms were identified regardless of the type of analysis. In general, these 13 GO terms could hardly be related to either the neurological or infection context. However, some of the enriched GO terms have been described in connection with viral infection. Polyoma virus infected cells showed an upregulation of genes associated with positive regulation of cell proliferation (GO:0008284) [50]. The term GO:0006977 (DNA damage response, signal transduction by p53 class mediator resulting in cell cycle arrest) is enriched in in neoplastic cells infected with Epstein-Barr Virus (EBV), another member of the herpesvirus family [51]. If only network meta-analysis was performed, GO:0006915 (apoptosis) was selected for example. The term GO:0006915 was identified to be overrepresented in retinal epithelium cells after infection with West Nile virus compared to uninfected cells [52]. In contrast, if only the merged data were analysed, the term GO:0007049 (cell cycle) was selected, which was detected to be enriched among differentially expressed genes in patients with EBV associated infectious mononucleosis [53].

### Methodical issues

We have demonstrated in a simulation study and by the analysis of a real-world example that network meta-analysis is a useful tool to make additional inferences from multiple independent studies with high-dimensional molecular expression data. While the results of network meta-analysis are highly correlated with the results of merged data analysis in simply study networks, network meta-analysis showed a higher correlation with the true fold changes than merged data analysis when one edge of the network was supported by multiple independent studies. This might indicate that the step of batch effect removal does not work well in the latter case. In our data analysis we used the ‘ComBat’ method to remove batch effects, and we used the same model for generating the simulation data. Thus, our results could be too optimistic with respect to the performance of the approach of analyzing the merged data. In practice, there may also be other types of batch effects which are not considered by the ‘ComBat’ model. Another batch effect removal approach, ‘FAbatch’, was proposed by Hornung et al. [54] that failed in their evaluation only in the case of extremely outlying batches or in cases where batch effects were very weak compared to the biological signal. Hornung et al. also provide a more detailed discussion on different batch effect models and methods for batch effect removal. These specific cases where batch effect removal fails are also an argument in favor of the network meta-analysis approach. Furthermore, as mentioned in the introduction, batch effect removal might also be a critical step when expression data was taken by different platforms.

In order to further study the issue of multiple batches we generated principal component plots under the simple and under the more complex study network scenarios, each before and after the step of batch effects removal (Additional file 3). Therein, samples of the control groups cluster together after batch effect removal, but samples from the different disease groups form separate clusters that may be represented by different batches. While most methods for batch effect removal have been devised for scenarios with dichotomous target variables (e.g. control versus diseased), typical scenarios of study networks involve multiple groups of different diseases, and these may be represented by different batches. By circumventing the step of batch effect removal, network meta-analysis can provide a helpful alternative over the analysis of merged data sets when there is uncertainty regarding the performance of the batch removal step.

Regarding the number *G* of genes involved in the analysis, we mentioned in the methods part that genes that are not involved in all studies of the network will be dropped from the analysis. In the case of larger study networks and when using network meta-analysis it would be easily possible to study sub-networks and thus to re-include some of the omitted genes. When using the data merging approach, studying sub-networks with some of the omitted genes re-included would require to newly perform the data merging with batch effect and normalization steps which would in summary make the results from the different sub-networks hard to compare.

Regarding the method for network meta-analysis, we have so far only used the methods by [2], implemented in the R-package ‘netmeta’. A comparison with the results of other network meta-analysis approaches would be an interesting addition which we intend for our future research.

## Additional files

Additional file 1 Example R-code for network meta-analysis. R-code that demonstrates how fold changes and their standard errors as obtained from ‘limma’ are used for network meta-analysis in the ‘netmeta’-R-package. (R 3 kb)

Additional file 2 Correlation between true and estimated logFC (Simulation no. 1a). Boxplots representing the correlation between true and estimated logFC versus sample size per group observed in the analysis of merged data and in network meta-analysis. 1000 simulation runs of two independent studies were performed with samples of *n*=10 per group (Simulation no. 1a). (PNG 6 kb)

Additional file 3 Principal component plots of merged data before and after batch effect removal. PCA plots of samples within a simple study network (top) or more complex study network (bottom) before and after batch effect removal. After batch effect removal the samples of the control groups cluster together. (PDF 136 kb)
