# Supplementary figures and images for: Network meta-analysis correlates with analysis of merged independent transcriptome expression data
Source: BMC Bioinformatics. 2019 Mar 15;20:144. doi: 10.1186/s12859-019-2705-9 (PMC6420731; doi:10.1186/s12859-019-2705-9)

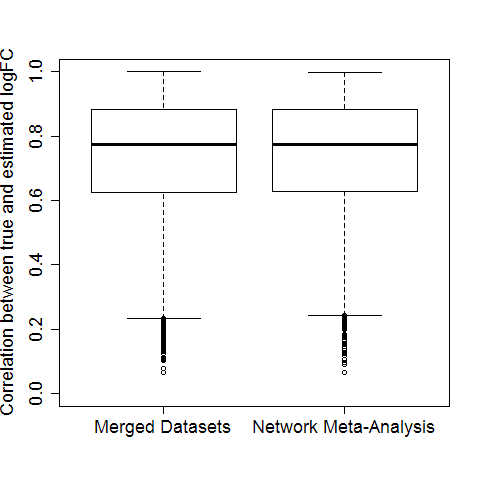

Supplement: Supplementary file 2 — Correlation between true and estimated logFC (Simulation no. 1a). Boxplots representing the correlation between true and estimated logFC versus sample size per group observed in the analysis of merged data and in network meta-analysis. 1000 simulation runs of two independent studies were performed with samples of n=10 per group (Simulation no. 1a). (PNG 6 kb) [file 12859_2019_2705_MOESM2_ESM.png]
