# Supplementary material for: Network meta-analysis correlates with analysis of merged independent transcriptome expression data
Source: BMC Bioinformatics. 2019 Mar 15;20:144. doi: 10.1186/s12859-019-2705-9 (PMC6420731; doi:10.1186/s12859-019-2705-9)
Supplement: Supplementary file 3 — Principal component plots of merged data before and after batch effect removal. PCA plots of samples within a simple study network (top) or more complex study network (bottom) before and after batch effect removal. After batch effect removal the samples of the control groups cluster together. (PDF 136 kb) [file 12859_2019_2705_MOESM3_ESM.pdf]

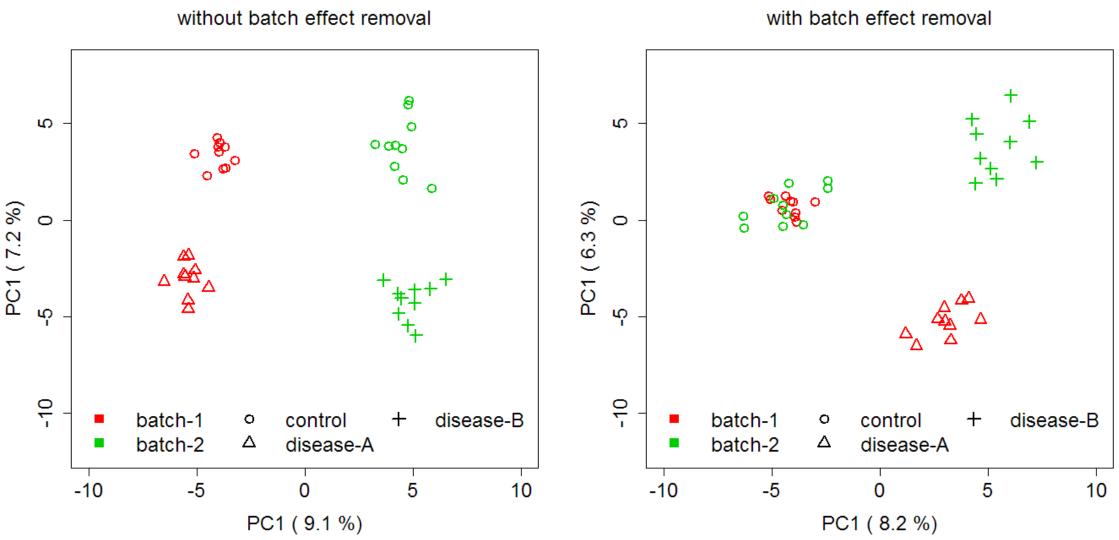

PCA plots of samples within a simple study network before and after batch effect removal. After batch effect removal the samples of the control groups cluster together.

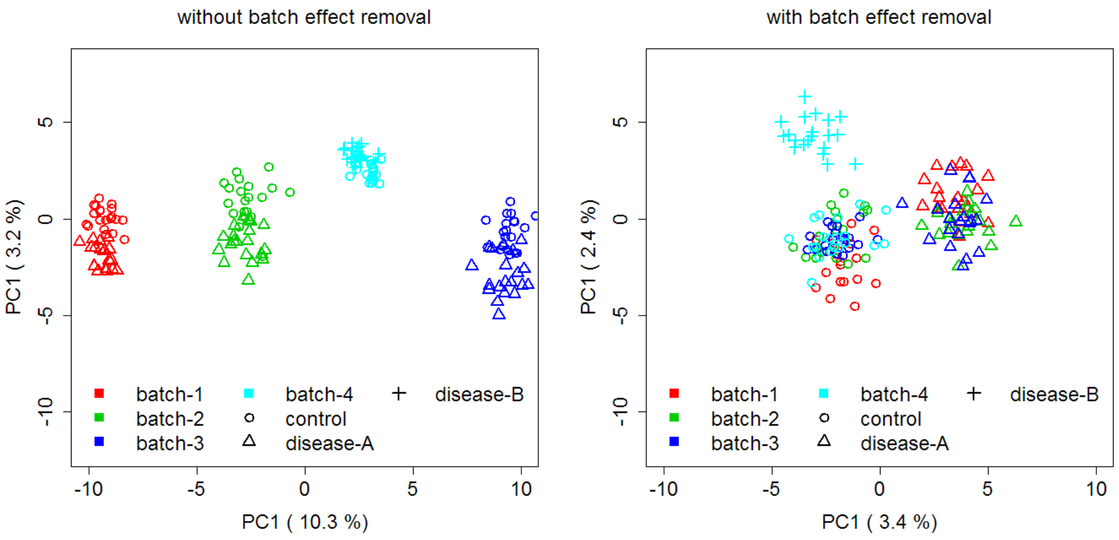

PCA plots of sample within a more complex study network before and after batch effect removal. After batch effect removal the samples of the control groups as well as the samples for disease A cluster together.
